# Fimasartan reduces neointimal formation and inflammation after carotid arterial injury in apolipoprotein E knockout mice

**DOI:** 10.1186/s10020-019-0095-0

**Published:** 2019-07-15

**Authors:** Jong-Ho Kim, I-Rang Lim, Hyung Joon Joo, Chi-Yeon Park, Seung-Cheol Choi, Han Saem Jeong, Soon Jun Hong

**Affiliations:** Department of Cardiology, Cardiovascular Center, Korea University College of Medicine, Seoul, Republic of Korea

**Keywords:** Atherosclerosis, Angiotensin receptor blockers, Fimasartan, Neointimal hyperplasia, Regulatory T cell

## Abstract

**Background:**

The beneficial effects of angiotensin II type 1 receptor blockers (ARBs) on atherosclerosis have been demonstrated in numerous studies. We investigated the effects of fimasartan on reducing neointimal formation and systemic inflammation after carotid artery (CA) injury in Apolipoprotein E knockout (ApoE KO) mice.

**Methods:**

ApoE KO mice were randomly allocated to Group I (without CA injury), Group II (without CA injury + Fimasartan), Group III (CA injury), and Group IV (CA injury + Fimasartan). Fimasartan was orally administered everyday starting 3 days before iatrogenic left CA injury.

**Results:**

At 28 days, neointimal hyperplasia and the inflammatory cytokines including TNFα, IL-6, ICAM, and MMP-9 in the peripheral blood were significantly reduced in Groups II and IV compared to Groups I and III, *respectively*.

All fimasartan-administered groups revealed significant increases of CD4^+^CD25^+^Foxp3^+^ regulatory T (Treg) cells with increased plasma levels of IL-10 and TGFβ. In addition, increased CD8^+^ T cells by fimasartan were correlated with reduced smooth muscle cell (SMC) proliferation in the neointima in Groups II and IV. Furthermore, the populations of Treg and CD8^+^ T cells in total splenocytes were increased in Groups II and IV compared to Groups I and III, *respectively*. The enlargement of spleens due to CA injury in the Group III was attenuated by fimasartan, as shown in the Group IV. These data indicate that fimasartan significantly reduced SMC proliferation in neointima and increased Treg cells in ApoE KO CA injury mice.

**Conclusions:**

This study suggests fimasartan could be an efficient strategy for reduction of atherosclerotic progression, with a decrease in immune response and systemic inflammation.

**Electronic supplementary material:**

The online version of this article (10.1186/s10020-019-0095-0) contains supplementary material, which is available to authorized users.

## Introduction

Angiotensin II (Ang II) is a pivotal component of the renin-angiotensin system (RAS) and is mainly regulated by Ang II type 1 (AT1) receptors. Activation of AT1 receptors by Ang II not only regulates blood pressure, but affects blood vessel inflammation and endothelial dysfunction in the progression of hypertension and atherosclerosis (Mehta and Griendling 2007). AT1 receptor blockers (ARBs) have been developed to selectively block AT1 receptors and reported to reduce progression of these diseases (Arishiro et al. 2007; Matsumura et al. 2011). Fimasartan, a newly developed ARB, is a Losartan analogue that has longer duration, and higher binding affinity and inhibitory activities than Losartan (Kim et al. 2012). In aortic balloon injury rabbit models, treatment with fimasartan attenuated plaque formation and disruption, and decreased macrophage accumulation in the plaques compared to the placebo group (Lee et al. 2013). A recent clinical study revealed that mild-to-moderate hypertensive patients who were administered fimasartan showed significant reduction in blood pressure compared to those administered Losartan (Lee et al. 2012).

Atherosclerosis, a main cause of cardiovascular disease, is a multifactorial process involving damage to endothelium, inflammation, lipid accumulation, and cell death. In addition, abnormal proliferation and migration of smooth muscle cells (SMCs) are attributed to triggering of the atherosclerotic plaque formation (Hansson and Libby 2006). Monocytes are also recruited to the atherosclerotic lesion and transformed into macrophages or foam cells with lipids (Weber et al. 2008). Moreover, interleukin (IL)-6, the proinflammatory cytokine, is produced by cells in atherosclerotic lesions and stimulates SMC proliferation and recruitment of inflammatory cells (Schuett et al. 2009). During atherogenesis, matrix metalloproteinase (MMP)-2 and MMP-9 are the main proteases, and their proteolytic activities increased in advanced atherosclerotic lesions (Wagsater et al. 2011). MMP-9 deficiency significantly reduced intimal plaque volume and length after temporary ligation of carotid arteries in apolipoprotein E knockout (ApoE KO) mice (Choi et al. 2005).

T cells are critical regulators of inflammatory responses and immune activities including atherosclerosis. Among various T cell subtypes, CD4^+^CD25^+^Foxp3^+^ regulatory T (Treg) cells have been reported to have atheroprotective effects by producing inhibitory cytokines such as IL-10 and transforming growth factor β (TGFβ) (Meng et al. 2016). Injection of Treg cells secreting high levels of IL-10 reduced atherosclerotic plaques and inflammation in ApoE KO mice (Mallat et al. 2003). Disruption of TGFβ signaling increased the progression of atherosclerosis and the accumulation of lipid and macrophages in plaques (Robertson et al. 2003). Moreover, patients with vulnerable coronary atherosclerotic plaques showed a significantly decreased number of Treg cells and IL-10 levels in plasma, and no significant difference in the plasma level of other cytokines or hormones (IL-6, IL-12, adiponectin, and resistin) when compared to stable patients (George et al. 2012). In addition, cytotoxic CD8^+^ T cells promoted the apoptosis of SMCs in atherosclerotic plaques via interactions between Fas/Fas ligands (Henderson et al. 1999). Adoptive transfer of CD8^+^ T cells significantly attenuated neointimal formation after arterial injury due to their lytic and cytotoxic activity against SMCs (Dimayuga et al. 2011).

ApoE KO mice have been popularly used to investigate the pathology and mechanisms of atherosclerosis because of their propensity to develop atherosclerotic plaques with a standard chow diet (Meir and Leitersdorf 2004). Therefore, based on the existing evidence, we investigated the effects of oral administration of fimasartan on neointimal formation and systemic inflammation after carotid artery (CA) injury in ApoE KO mice.

## Materials and methods

### Drugs and administration

**Fig. 1:**
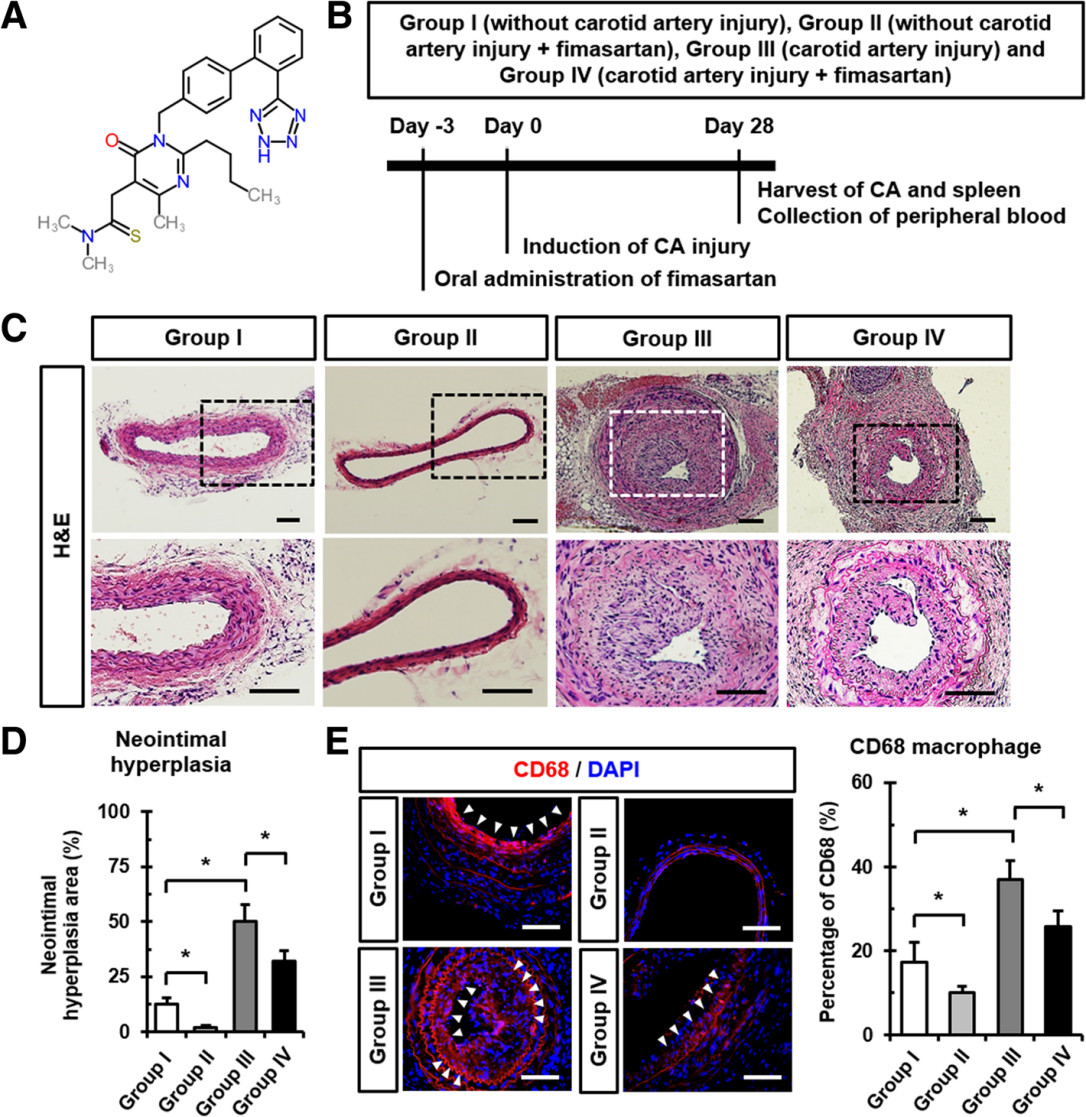
Regression of CA injury-induced neointimal hyperplasia in ApoE KO mice by Fimasartan administration. **a** Chemical structure of fimasartan (2-n-butyl-5-dimethylamino-thiocarbonyl-methyl-6-methyl-3-{[2-(1H-tetrazole-5-yl)biphenyl-4-yl]methyl}pyrimidin-4(3H)-one). **b** Schematic depiction of the study protocol. **c** Representative H&E-stained images of the CA cross sections of ApoE KO mice. Boxed regions were magnified in the under panels. Scale bar: 100 μm. **d** Quantitative analysis of the incidence of neointimal hyperplasia areas in CA after CA injury and/or fimasartan administration. Data shown represent mean ± SD on 40 sections (two fields per section, two sections per CA, *n* = 10 for each group). *p < 0.05. **e** Representative immunofluorescence images and quantitative analysis results showing CD68^+^ macrophage (red) expression in CA at 28 days after CA injury and/or fimasartan administration. White arrows indicate CD68^+^ macrophages in CA. Nuclei were stained with DAPI. Scale bar: 100 μm. *p < 0.05. All experimental data are from *n* = 10 of ApoE KO mice in each group

Fimasartan (Kanarb; BR-A-657; C_27_H_30_N_7_SK_3_H_2_O) was obtained from Boryung Pharmaceutical Co. Ltd. (Seoul, Republic of Korea) (Fig. 1a). Fimasartan was prepared by mixing and emulsifying it in distilled water (dW). Fimasartan was administered via oral gavage starting 3 days before CA injury at a dose of 3 mg/kg per day. This suspension was given daily for 28 days.

### Animal experimental design

All procedures were approved by the Korea University Institutional Ethics Committee for Animal Research (KUIACUC-2015-53 and KUIACUC-2016-7). All animals were maintained in a specific pathogen-free (SPF) grade animal room. All studies complied with the guidelines from the US National Institutes of Health and Directive 2010/63/EU of the European Parliament on the protection of animals used for scientific purposes.

ApoE KO mice were purchased from Orient Experimental Animal Laboratory {Gyeonggi, Republic of Korea; living modified organisms (LMO) registration number: LMI14–137}. All ApoE KO mice were fed with normal chow diet. The mice were randomly assigned to the following four groups: Group I (without CA injury; *n* = 20), Group II (without CA injury + Fimasartan administration; *n* = 21), Group III (CA injury; *n* = 25), and Group IV (CA injury + Fimasartan administration; *n* = 24) (Fig. 1b). Sample size was determined assuming an effect size f of 0.4 to yield a study power of 80% with one-sided type I error rate of 0.05. A total of 76 mice in 4 groups (minimal *n* = 19 per group) was calculated. Considering 25% drop-out rate for surgical procedure, 25 mice were randomized to Group III and IV.

### Induction of carotid artery injury

Ninety male ApoE KO mice weighing 24–26 g were anaesthetized by intraperitoneal (IP) injection of a mixture of ketamine (80 mg/kg; Yuhan) and xylazine hydrochloride (8 mg/kg; Rompun, Bayer). Adequacy of anesthesia was monitored continually by pinching the toe and respiration. Left common carotid artery (CA) injury was performed under a dissecting microscope. After midline neck incision, the left external CA was tied off distally, and a 0.014 in. flexible angioplasty guide wire was advanced 1 cm through transverse arteriotomy of the external CA. Endothelial denudation was achieved by making three rotational passes along the common CA. After induction of CA injury, muscle layers and skin were closed with 6–0 silk sutures. All ApoE KO mice were subcutaneously (SC) injected with an analgesic agent, ketoprofen (5 mg/kg) after surgery and monitored at least twice a day for the first 7 days and at least once a day until 28 days after surgery. For sacrifice, ApoE KO mice were euthanized by IP injection with a mixture of ketamine (80 mg/kg) and xylazine hydrochloride (8 mg/kg).

### Plasma lipid measurement

At 28 days, plasma high-density lipoprotein (HDL) and low-density lipoprotein (LDL)/ Very-LDL (VLDL) cholesterol were separated and measured using HDL and LDL/VLDL cholesterol assay kit (ab65390, Abcam) in addition to total cholesterol (TC). Plasma triglyceride (TG) levels were also analyzed using triglyceride quantification assay kit (ab65336, Abcam). The absorbance of each assay product was measured using a microplate reader (M2e, Molecular Devices).

### Tissue preparation

After sacrifice, CA and spleen tissues of ApoE KO mice were perfusion-fixed under continuous pressure with 4% paraformaldehyde (PFA; P6148, Sigma-Aldrich), and embedded in paraffin. The embedded CA and spleen tissues were then sectioned into 5 or 10 μm-thickness serial cross sections.

### Histological and immunohistochemical staining

Paraffin-embedded sections of CA and spleen tissues were stained with hematoxylin and eosin (H&E) using hematoxylin (HHS32) and eosin Y solution (HT110316, both from Sigma-Aldrich) according to the Harris modified method. The tissue sections were then mounted with VectaMount mounting medium (H-5000, Vector). The percentage of neointimal hyperplasia was calculated by subtracting the area of vessel lumen from the area of internal elastic lamina.

For immunofluorescence staining, the CA and spleen tissue sections were treated with Proteinase K (PK; 21627, Merck Millipore), and blocked with 5% normal goat serum (NGS; #16210, Invitrogen). Then, the tissue sections were incubated with primary antibodies against Alexa Fluor 488-conjugated CD4 (100,423, Biolegend) and CD68 (sc-9139, Santa Cruz). The tissue sections were subsequently stained with Alexa Fluor 594- (A11012) or 488-conjugated anti-rabbit antibodies (A21441, both from Molecular Probes) and then incubated with 4′,6-diamidino-2-phenylindole (DAPI; D9542, Sigma-Aldrich). The tissue sections were then mounted with fluorescence mounting medium (S3023, DAKO).

Staining of CA sections with 3,3′-Diaminobenzidine (DAB) was performed with primary antibodies for CD4 (14–0041), CD8 (14–0081, both from eBioscience), and smooth muscle α-actin (α-SMA; A2547, Sigma-Aldrich). After immunostaining, the sections were incubated with biotinylated anti-mouse IgG antibodies for 30 min at room temperature (RT), followed by treatment with ABC reagent (PK-6100, Vector). After sufficient color development with DAB substrate (SK-4100, Vector), the tissue sections were then mounted with VectaMount mounting medium.

All tissue sections were randomly selected for analysis. All digital images were obtained using a TE-FM Epi-Fluorescence system attached to an Olympus BX61 inverted microscope. Images of sections were photographed at a magnification of X100 or X400 and analyzed by a trained, blinded reader. All quantification was achieved using Image-Pro software (Ver. 7.0, Media cybernetics).

### Enzyme-linked immunosorbent assay (ELISA)

Peripheral blood samples of ApoE KO mice were collected from the postcaval vein using a 24-gauge catheter (382412, BD) at 28 days. The blood plasma was separated using Ficoll-Paque plus (#17–1440-03, GE). Circulating IL-6 (M6000B), TGFβ (MB100B), IL-10 (M1000B), tumor necrosis factor (TNF) α (MTA00B), intercellular adhesion molecule (ICAM; MIC100), MMP-9 (MMPT90), vascular cell adhesion molecule (VCAM; MVC00), and MMP-2 (MMP200, all from R&D systems) levels were evaluated using mouse ELISA kits. The ELISA reaction product was quantified by measuring absorbance at 450 nm and 540 nm using a microplate reader (iMark), and data were analyzed using Microplate Manager software (Ver. 6.1, both from Bio-rad).

### Flow cytometry

To prepare samples for flow cytometric analyses, peripheral blood was drawn into heparinized-tubes after 28 days following CA injury. Peripheral blood mononuclear cells (PBMNCs) were isolated immediately using Ficoll-Paque Plus (GE). Splenocytes were isolated from the minced spleen tissues. After adding PBS, the samples were filtered with 100 μm nylon meshes, washed with RPMI (12-702F, Lonza) containing fetal bovine serum (FBS; #16000–044, Invitrogen), and treated with red blood cell lysis buffer containing NH_4_Cl, KHCO_3_, and EDTA.

PBMNCs and splenocytes were subsequently incubated for 20 min at RT with the following primary antibodies: fluorescein isothiocyanate (FITC)-conjugated IgG isotype control (11–4011), phycoerythrin (PE)-conjugated IgG isotype control (12–4012, both from eBioscience), Allophycocyanin (APC)-conjugated IgG isotype control (400119), FITC-conjugated CD4 (100406), FITC-conjugated CD8 (100705), or APC-conjugated CD25 (102012, all from Biolegend). To detect the intracellular Foxp3, PBMNCs and splenocytes were then fixed and permeabilized using Foxp3 Fix/Perm buffer (421403, Biolegend) according to the manufacturer’s recommendations, and stained with PE-conjugated Foxp3 (12–5773, eBioscience). For negative control experiments, all conditions were kept the same except that IgG isotype controls were used. After washing twice with PBS + 2% FBS, 5 X 10^4^ PBMNCs and 10 X 10^4^ splenocytes from each sample were analyzed on a FACS Calibur flow cytometer using Cell Quest Pro software (both from BD).

### In vitro T cell proliferation assay

CD3^+^ T cells were isolated from splenocytes of male ApoE KO mice by MACS (Miltenyi Biotec) using PE anti-mouse CD3 antibody (100205, Biolegend) and anti-PE microbeads (130–105-639, Miltenyi Biotec). CD3^+^ T cells were stained with carboxyfluorescein diacetate, succinimidyl ester (CFDA SE; V12883, Invitrogen) to label the proliferating cells. CD3^+^ T cells were then seeded with fimasartan (1 mM) in RPMI containing 5% FBS. After 5 days, T cell proliferation was analyzed by measuring CFDA SE signal of 10 X 10^4^ cells from each sample using flow cytometry (MACS Quant, Miltenyi Biotec).

### T cell lytic activity assay

CD8^+^ T cells were sorted from splenocytes of male ApoE KO mice by MACS using CD8^+^ T cell isolation kit (130–104-075, Miltenyi Biotec). Mouse aortic smooth muscle cells (mSMCs; C57–6080, Cell Biologics) were labeled with CFDA SE. mSMCs were co-cultured at a CD8^+^ T cell to mSMC ratio of 1:1 or 3:1 in DMEM (SH30021, Hyclone) containing 5% FBS. For measuring basal lysis of mSMCs, the cells were incubated without CD8^+^ T cells. After 4 h of co-culture, attached cells were washed with PBS and phase contrast, and fluorescent images were taken. The cells were then trypsinized and stained with propidium iodide (PI; 556463, BD Biosciences) for analysis using flow cytometry (MACS Quant, Miltenyi Biotec). Percentages of mSMC lysis were calculated using results of flow cytometry as previously described (Dimayuga et al. 2011).

### Statistical analysis

All statistical values were expressed as mean ± standard deviation (SD). Analysis of variance (ANOVA) was used to compare normally distributed data from all groups. Significant differences between means were determined using ANOVA followed by the Student-Newman-Keuls test. All *P*-values are two-sided, and values of *p < 0.05 were considered statistically significant. All statistical analyses were performed using Sigma Stat software (Ver. 3.1, Systat Software).

## Results

### Fimasartan administration suppresses neointimal hyperplasia after CA injury in ApoE KO mice

Neointimal formation in CA was significantly increased by mechanical injury in the Group III (50.06 ± 7.50%) compared to the Group I (12.31 ± 2.97%). Fimasartan administration significantly reduced neointimal formation in Groups II (2.30 ± 0.66%) and IV (32.03 ± 4.69%) compared to Groups I and III, *respectively* (Fig. 1c and d). In atherosclerotic lesions of CA, CD68^+^ macrophages, a main plaque component, accumulated after CA injury. Fimasartan-administered Group IV (25.89 ± 43.77%) showed reduced expression of CD68^+^ macrophages in CA compared to the Group III (36.97 ± 4.65%; Fig. 1e). Therefore, mechanical CA injury increased neointimal hyperplasia, and fimasartan attenuated the neointimal formation and accumulation of macrophages in ApoE KO mice. Moreover, fimasartan administration and CA injury had no significant effects on the plasma lipid levels with respect to triglyceride, HDL, LDL/VLDL, and total cholesterol (Additional file 1: Table S1).

### Fimasartan administration increases Treg cells and anti-atherosclerotic cytokines in ApoE KO mice

**Fig. 2:**
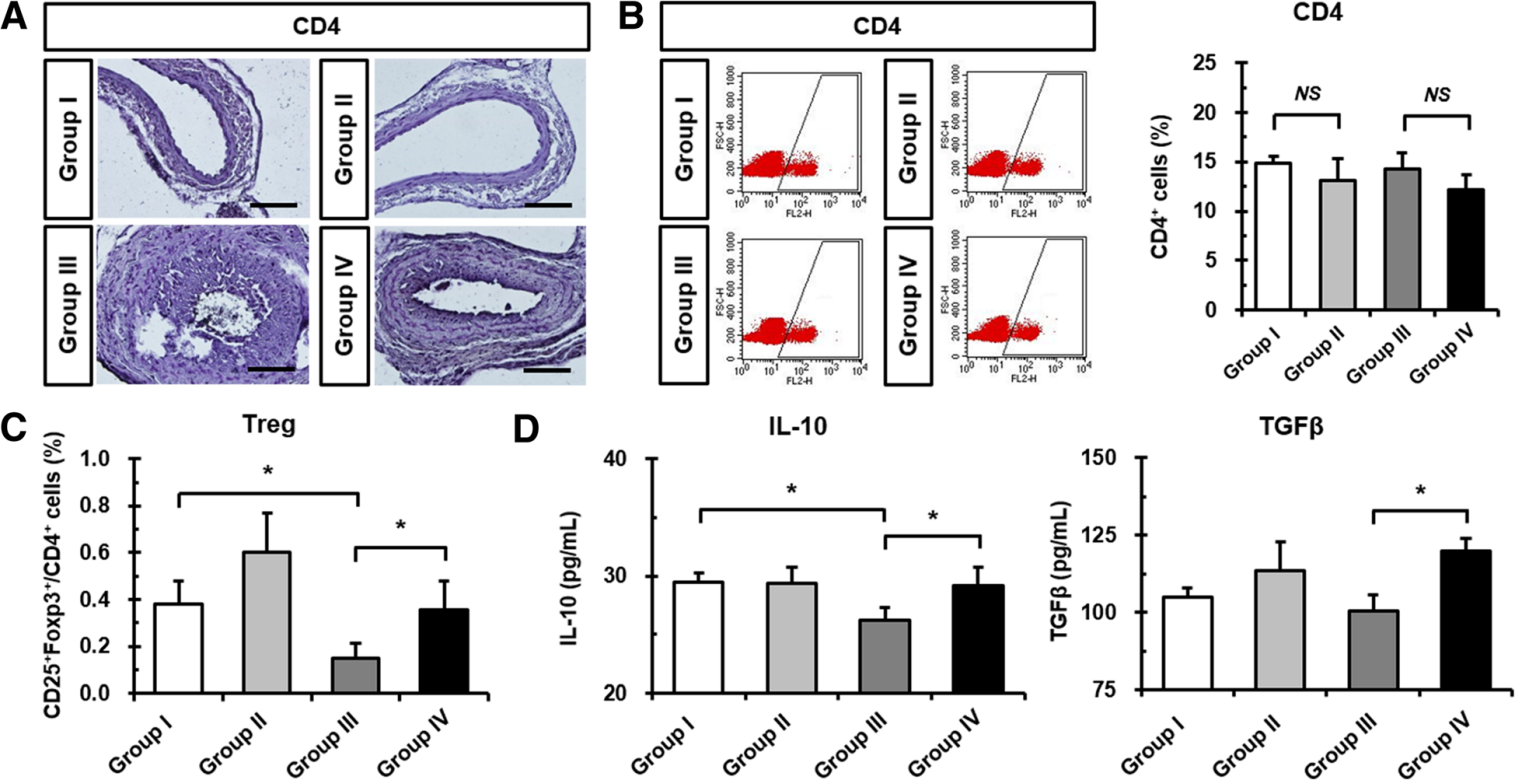
Effects of Fimasartan on CD4^+^ T cells, Treg cells, and anti-inflammatory cytokines in the peripheral blood of ApoE KO mice. **a** Immunohistochemical images showing the expression of CD4 (brown) at 28 days after CA injury and/or fimasartan administration. Scale bar: 100 μm. **b** Representative pictures showing that CD4^+^ T cell subsets were gated using flow cytometry. The numbers indicate positive percentages of CD4^+^ T cells. **c** Results of statistical analyses of Treg cells in peripheral blood at 28 days after CA injury and/or fimasartan administration. **d** Plasma levels of Treg-related cytokines, including IL-10 and TGFβ were measured by ELISA. All experimental data are from *n* = 12 of ApoE KO mice in each group. The values are presented as the mean ± SD. *p < 0.05. *NS*, not significant

To investigate the effects of mechanical CA injury and fimasartan administration on CD4^+^ T cells, we performed immunohistochemical staining of CD4 in CA sections. No differences were evident among the four groups with respect to infiltration of the atherosclerotic plaque (Fig. 2a). Circulating CD4^+^ T cells were slightly decreased in all fimasartan-administered Groups II and IV, but not significantly compared to Groups I and III, *respectively* (Fig. 2b). Furthermore, we investigated the effects on the specific subset of CD4^+^ T cells that are CD4^+^CD25^+^Foxp3^+^ Treg cells by CA injury and/or fimasartan administration. The fimasartan-administered Group II (0.60 ± 0.17%) showed increased Treg cells in peripheral blood compared to the Group I (0.38 ± 0.10%) (Fig. 2c). We found that the populations of Treg cells in the Group III (0.15 ± 0.06%) were significantly reduced in the peripheral blood compared to the Group I. A reduced population of Treg cells due to CA injury (Group III) was significantly increased in the Group IV (0.36 ± 0.13%) by fimasartan administration (Fig. 2c). Furthermore, cytokine expressions secreted by Treg cells including IL-10 and TGFβ demonstrated a tendency to be similar to levels of Treg cells in the peripheral blood (Fig. 2d). The IL-10 level in plasma was significantly decreased in the Group III (26.18 ± 1.14 pg/mL) due to CA injury compared to the Group I (29.48 ± 0.78 pg/mL). The decreased IL-10 level in injured the Group III was significantly increased in the Group IV (29.20 ± 1.56 pg/mL) that received fimasartan. Similarly, the TGFβ level in plasma was also significantly increased by fimasartan administration in spite of CA injury (Group III vs. Group IV; 100.54 ± 5.25 pg/mL vs. 119.77 ± 4.35 pg/mL). Taken together, fimasartan administration increased atheroprotective Treg cells and related cytokines.

### Fimasartan administration reduces SMC proliferation and inflammatory cytokines/chemokines after CA injury

**Fig. 3:**
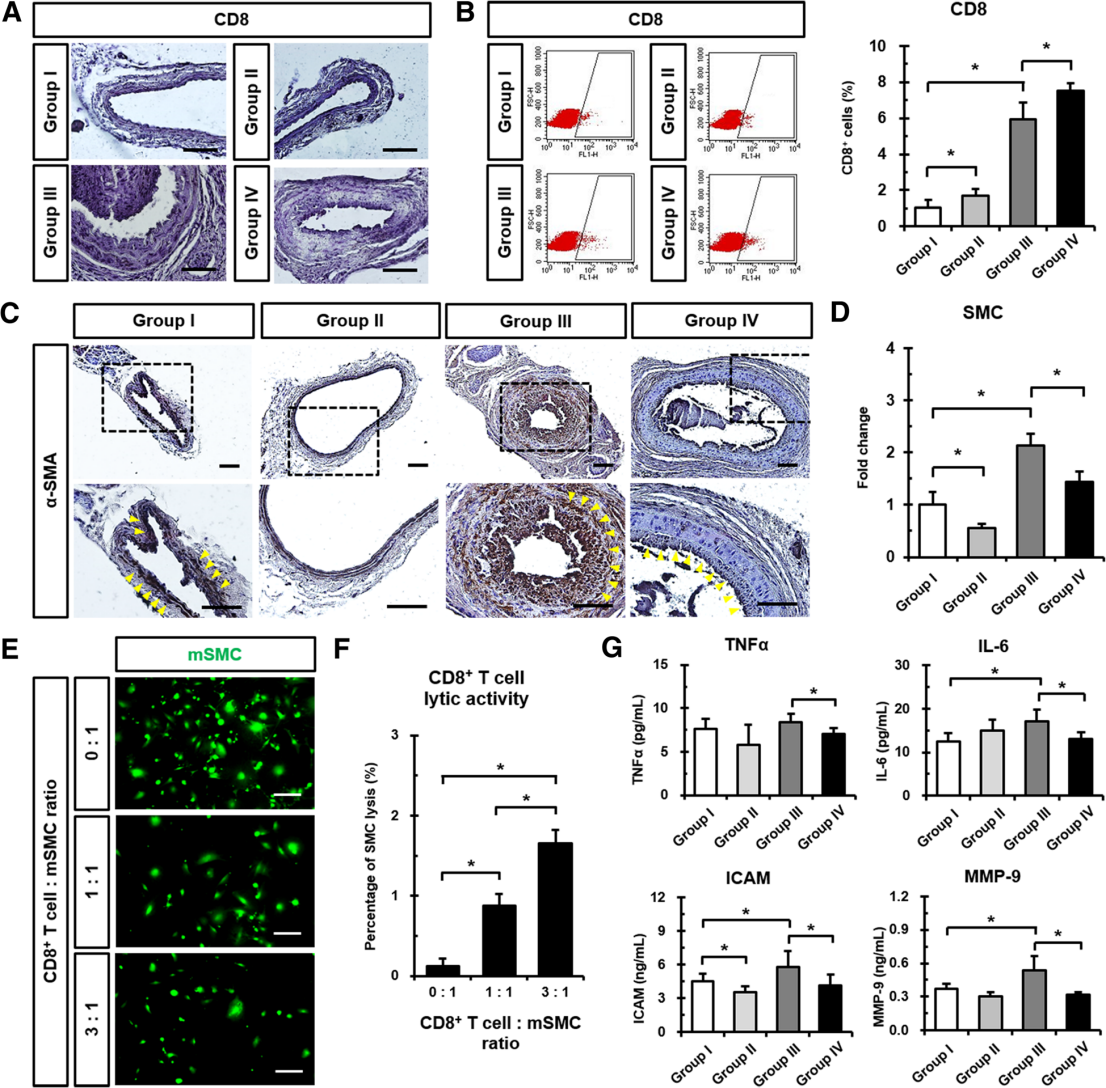
Effects of Fimasartan on CD8^+^ T cells, SMCs, and cytokines/chemokine expression after CA injury. **a** Immunohistochemical images showing the expression of CD8^+^ T cells (brown) in CA at 28 days after CA injury and/or fimasartan administration. Scale bar: 100 μm. **b** Representative pictures showing that CD8^+^ T cell subsets were gated using Flow cytometry. Results of statistical analysis of CD8^+^ T cells in peripheral blood at 28 days after CA injury and/or fimasartan administration. **c** Representative immunohistochemical images of α-SMA^+^ expression in CA, showing reduced SMCs (brown) after fimasartan administration. Boxed regions were magnified in the under panels. Yellow arrows indicate α-SMA^+^ cells in CA. Scale bar: 100 μm. **d** Quantification of SMCs in CA at 28 days after CA injury and/or fimasartan administration. Data shown represent the mean ± SD on 48 sections (two fields per section, two sections per CA, *n* = 12 for each group). **e** Fluorescent images of CD8^+^ T cell lytic activity assay against SMCs. CFDA SE-labeled mSMCs were co-cultured for 4 h at a CD8^+^ T cell to mSMC ratio of 0:1, 1:1 or 3:1. **f** CD8^+^ T cell lytic activity assay against SMCs examined by flow cytometry. Quantification of PI-positive and CFDA SE-labeled mSMCs. **g** The plasma levels of inflammatory cytokines/chemokines (TNFα, IL-6, ICAM, and MMP-9). All experimental data are from *n* = 12 of ApoE KO mice in each group. Values are presented as the mean ± SD. *p < 0.05. *NS*, not significant

Since the infiltration of T cells into plaques is highly involved in the development of neointima and atherosclerotic plaque, we examined whether mechanical CA injury and fimasartan administration affected the population of cytolytic CD8^+^ T cells. Immunohistochemical staining of CA sections revealed that CD8^+^ T cells infiltrating the neointima were augmented (Fig. 3a), and circulating CD8^+^ T cells also were increased in the Group III due to CA injury as assessed by flow cytometry. All fimasartan-administered groups showed significantly increased CD8^+^ T cells in the peripheral blood compared to non-administered groups. Circulating CD8^+^ T cells significantly increased in the Group IV (7.51 ± 0.45%), which was given fimasartan compared to the Group III (5.92 ± 0.96%) (Fig. 3b). Moreover, we observed the robust expression of α-SMA^+^ SMCs in the neointima of the Group III (2.14 ± 0.22) after CA injury compared to the Group I (1.00 ± 0.24). The Group IV (1.44 ± 0.19) demonstrated significantly reduced expression of SMCs, which is associated with a significant increase in CD8^+^ T cells by fimasartan administration compared to the Group III (Fig. 3c and d). To examine CD8^+^ T cell-mediated reduction of neointimal hyperplasia, mSMCs were co-cultured with splenic CD8^+^ T cells obtained from ApoE KO mice. After 4 h of co-culture, CFDA SE-stained mSMCs decreased in the presence of CD8^+^ T cells (Fig. 3e). Indeed, PI-positive mSMCs increased significantly when they were co-cultured with CD8^+^ T cells at the 1:1 and 3:1 ratio (0.88 ± 0.15% and 1.65 ± 0.17%) compared to no T cells (0.12 ± 0.10%; Fig. 3f). Therefore, there were cytolytic activities of CD8^+^ T cells against mSMC.

After 28 days of CA injury, significantly higher plasma levels of inflammatory cytokines including IL-6, ICAM, and MMP-9 were observed in the Group III (17.11 ± 2.77 pg/mL, 5.79 ± 1.41 ng/mL, and 0.54 ± 0.13 ng/mL, *respectively*) than the Group I (12.50 ± 1.81 pg/mL, 4.51 ± 0.72 ng/mL, and 0.37 ± 0.05 ng/mL, *respectively*). Fimasartan administration significantly decreased TNFα (Group III vs. Group IV; 8.41 ± 0.93 pg/mL vs. 7.05 ± 0.34 pg/mL), IL-6 (vs. 13.06 ± 1.51 pg/mL), ICAM (vs. 4.18 ± 0.97 ng/mL), and MMP-9 (vs. 0.32 ± 0.02 ng/mL) in plasma (Fig. 3g). These results suggest that fimasartan administration reduced SMCs in the neointima, possibly via regulation of cytolytic CD8^+^ T cells, and decreased various inflammatory cytokines promoted by mechanical CA injury.

### Fimasartan administration suppresses splenomegaly and pathological immune response after CA injury in ApoE KO mice

**Fig. 4:**
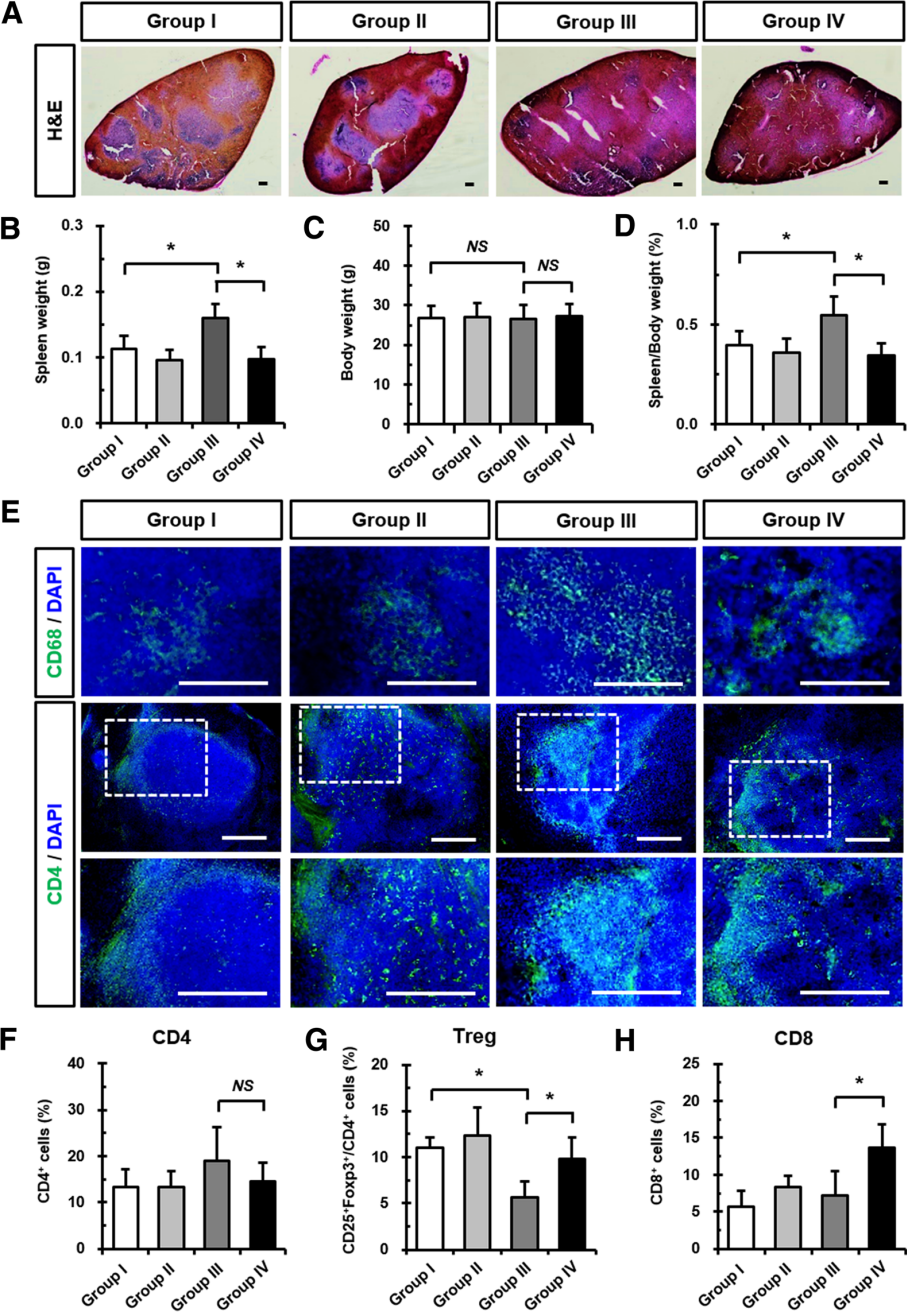
Changes in Fimasartan on splenomegaly and immune cells in spleens after CA injury. **a** Representative H&E-stained images showing the size and distribution of splenic pulp at 28 days after CA injury and/or fimasartan administration. Scale bar: 100 μm. **b-d** Quantitative analyses of relative spleen weight, body weight, and ratio of spleen weight/body weight were performed at 28 days after CA injury and/or fimasartan administration. **e** Representative immunofluorescence images showing CD68^+^ macrophages (green) and CD4^+^ T cells (green) expression in the spleen at 28 days after CA injury and/or fimasartan administration. Boxed regions were magnified in the under panels. Nuclei were stained with DAPI. Scale bar: 100 μm. **f-h** Flow cytometry analyses of the number of CD4^+^, Treg, and CD8^+^ T cells from spleen tissues at 28 days after CA injury and/or fimasartan administration. All experimental data are from *n* = 9, 10, 12, and 12 of ApoE KO mice in each group. The values are presented as the mean ± SD. *p < 0.05. *NS*, not significant

The spleen plays essential roles in inflammatory and immune responses, and the enlargement of spleens which is known as splenomegaly occurs in response to inflammation. After 28 days of mechanical CA injury and/or fimasartan administration, H&E-stained images revealed the incidence of splenomegaly in the Group III. In spite of the CA injury, fimasartan-administered Group IV showed a reduction in spleen size compared to the Group III (Fig. 4a). The changes in spleen and body weights were measured (Fig. 4b and c). Spleen/body weight ratio was significantly increased in the Group III (0.60 ± 0.08%) compared to the Group I (0.41 ± 0.08%), and significantly decreased in the Group IV (0.35 ± 0.07%) compared to the Group III (Fig. 4d).

Furthermore, the effects on T cells and macrophages in the spleen from CA injury and/or fimasartan administration were investigated. As shown in Fig. 4e, the Group III with elevated inflammatory response due to CA injury showed a relatively increased expression of CD68^+^ macrophages and CD4^+^ T cells in spleens compared to the Group I. A relatively reduced expression of splenic macrophages was detected in the Group IV compared to the Group III. Moreover, in vitro T cell proliferation assay revealed that proliferation of splenic CD3^+^ T cells increased significantly in the presence of fimasartan (Additional file 1: Figure S1). Therefore, we examined the changes in the subsets of splenic T cells 28 days after oral administration. The Group III (18.94 ± 7.39%) demonstrated a slightly increased population of CD4^+^ splenocytes compared to the Group IV (14.57 ± 4.04%), however, there was no significant difference (Fig. 4f). Intriguingly, the population of Treg splenocytes decreased in the Group III (5.61 ± 1.79%) compared to the Group I (11.07 ± 1.04%), suggesting that splenic Treg cells were reduced by CA injury (Fig. 4g). Additionally, all fimasartan-administered groups showed an increased population of splenic CD8^+^ T cells when compared to non-administered groups. In particular, splenic CD8^+^ T cells were significantly increased in the Group IV (6.80 ± 1.61%) compared to the Group III (3.57 ± 1.67%) (Fig. 4h). Consequently, these data indicate that mechanical injury of the CA induced the local inflammation leading to a systemic response, including the immune cells and organs that could be ameliorated by fimasartan administration.

## Discussion

The novelties of this study were (Mehta and Griendling 2007) the investigation of fimasartan in atherosclerotic and mechanical CA injury animal models, (Arishiro et al. 2007) examination of the effects of fimasartan on various immune cells, pro−/anti-inflammatory cells, and SMCs from the neointima to blood, and (Matsumura et al. 2011) observation of the effects of fimasartan on macrophages and immune cells including Treg cells in spleens.

Fimasartan is a ninth ARB, which was approved in Korea in 2010 and in 12 Latin American countries in 2014 for the treatment of hypertension. Fimasartan has a selective AT1 receptor antagonist effect without a partial agonistic effect of the angiotensin II receptor via insurmountable binding. It possessed rapid and potent antihypertensive effects (Chi et al. 2013). Additionally, preclinical studies showed anti-inflammatory and organ-protecting effects beyond its BP-lowering effect (Angeli et al. 2018).

We have started administering fimasartan 3 days before vascular injury in accordance with the previous study. According to the pharmacokinetics of fimasartan, plasma concentration gradually increases in 2 days (Lee et al. 2011) and reaches a plateau after that. The dose of 3 mg/kg of fimasartan was selected based on previous studies showing that administration of 12.5 mg/kg induced drug-related multiple organ failure, and the highest tolerated dose of fimasartan was 6 mg/kg (Lee et al. 2013; Kim et al. 2015). Numerous animal studies have reported that ARBs attenuate atherosclerotic progression and regulate immune responses including a decrease in macrophages (Matsumura et al. 2011; Meng et al. 2015; Johnstone et al. 2004). In the present study, mechanical injury of the CA increased neointimal formation and the accumulation of macrophages that were reduced by fimasartan administration (Fig. 1c-e). These findings suggest that fimasartan administration regulates neointimal formation and macrophage accumulation in plaques in atherosclerotic and mechanical CA injury animal models.

Previous studies have reported that T cells are critical in the development of hypertension, and especially Ang II increases the activation of T cells and production of proinflammatory cytokines (Hoch et al. 2009). Consistent with these reports, the current study demonstrated the tendency for decreasing CD4^+^ populations via oral administration of fimasartan that is one of ARBs (Fig. 2b). However, there were no significant differences because various subtypes of CD4^+^ T cells were mixed with the peripheral blood. Interestingly, fimasartan administration significantly elevated the population of Treg cells (Fig. 2c). In the coincidence with our study, Meng et al. showed that Valsartan administration in prolonged Ang II-treated ApoE KO mice significantly increased T helper cell type 2 (Th2) and Treg cells, and decreased Th1 and Th17 cells with proatherosclerotic properties (Meng et al. 2015). Treg cells have been shown to produce IL-10 and TGF-β, and their protective roles against atherosclerosis have been suggested in in vitro and in vivo studies (Robertson et al. 2003; Mallat et al. 1999). IL-10 deficient mice showed increased susceptibility in atherosclerotic lesion size and in vivo transfer of murine IL-10 reduced atherosclerotic lesion (Mallat et al. 1999). Secretion of IL-10 from Treg cells can promote a switch of M1 macrophages that secrete TNFα toward anti-atherogenic M2 macrophages (Spitz et al. 2016). Moreover, TGF-β is critical for Treg cell-mediated suppression, particularly in diminishing atherogenesis, and controls the development of thrombosis (Meng et al. 2016). Deletion of TGF-β enhanced the progression of atherosclerotic lesions and the accumulation of inflammatory cells (Robertson et al. 2003).

In the present study, the Group III showed increased CD8^+^ T cells in the peripheral blood due to mechanical injury-stimulated immune responses (Fig. 3b). In addition, we also observed increased CD8^+^ T cells by fimasartan and reduced SMCs in the neointima via CD8^+^ T cell-mediated cytolytic activities against SMCs (Fig. 3c-f). As is well known, cytotoxic CD8^+^ T cells have anti-atherosclerotic effects by reducing the proliferation of SMCs and neointimal formation through interactions between Fas/Fas ligands, inducing the lysis of SMCs (Henderson et al. 1999; Dimayuga et al. 2011). On the other hand, the effects of CD8^+^ T cells on atherosclerotic progression are still controversial. Several studies have reported that these effects could contribute to plaque rupture, thrombosis, or formation of aneurysms (Kyaw et al. 2013; Zhou et al. 2013). In our study, however, no rupture of plaques or thrombosis was observed.

Our study revealed that systemic inflammation decreased in fimasartan-administered and uninjured Group II compared to the Group I (Fig. 3), because proinflammatory transcription factors such as nuclear factor-kappa B (NF-κB) and activator protein-1 (AP-1) induced by Ang II were affected by fimasartan as an ARB. (Sanchez-Lemus et al. 2008) Moreover, fimasartan administration significantly decreased IL-6 level in the peripheral blood (Fig. 3g), which is reported to block the immunosuppressive effects of Treg cells (Pasare and Medzhitov 2003). The other proinflammatory cytokines including TNFα and ICAM were significantly reduced in the Group IV compared to the Group III. However, the Group IV (10.54 ± 2.36%) showed a tendency for decreasing VCAM levels compared to the Group III (12.33 ± 2.38%) (Additional file 1: Figure S2A). After 28 days of fimasartan administration and/or CA injury, MMP-2 (Additional file 1: Figure S2B) and MMP-9 were decreased in the Group IV compared to the Group III, that MMPs are involved in degrading the extracellular matrix (ECM) and destabilizing the atherosclerotic cap (Newby 2005; Yabluchanskiy et al. 2013).

Changes in spleens after fimasartan administration and/or mechanical CA injury were investigated for the first time in the current study. The spleen is a pivotal organ for immune cells and the inflammatory cytokines that are involved in the development of atherosclerosis (Potteaux et al. 2015). Splenectomized ApoE KO mice had significantly larger atherosclerotic lesions in the aortic root than the sham group, indicating an atheroprotective role of the spleen (Rezende et al. 2011). As shown in Fig. 4, CA-injured mice revealed splenomegaly, and the increased size of spleens was reduced after 28 days of fimasartan administration. Our results corroborate a previous study finding that peripheral administration of LPS augmented inflammatory responses with higher proinflammatory cytokine levels such as TNF-α, IL-1β, and IL-6 in blood. Also, administration of Candesartan efficiently blocked AT1 receptors in the red pulp of rat spleens and decreased LPS-induced increases of proinflammatory cytokine levels (Liverani et al. 2014; Sanchez-Lemus et al. 2009). In addition, we demonstrated that fimasartan reduced the size of pulps, splenic macrophages, and CD4^+^ T cells in CA-injured mice with ameliorated atherosclerosis (Fig. 4a-e). Splenic Treg cells also significantly increased by fimasartan (Fig. 4g) that may be attributed to immune and inflammatory responses, and systemic circulation of various pro−/anti-inflammatory cytokines and immune cells.

This study has a few limitations. The effects on other cytokines/chemokines related to the development of neointima and atherosclerotic plaque by fimasartan administration and CA injury need to be elucidated (Tao et al. 2016). Additional studies on the effects of fimasartan administration on other leukocytes involved in atherosclerosis would support anti-atherogenic effects of ARBs (Weber et al. 2008).

## Conclusion

Our study elucidated the positive effects of fimasartan in regulation of immune and inflammatory responses using a mechanical CA injury animal model for the first time. Our findings suggest that fimasartan reduced the neointimal hyperplasia and increased Treg cells with higher IL-10 and TGF-β levels that have anti-atherosclerotic properties. Furthermore, fimasartan efficiently reduced increased SMCs in the neointima after mechanical CA injury via the cytolytic activity of CD8^+^ T cells. We also showed that fimasartan mitigated CA injury-induced splenomegaly and increased the atheroprotective Treg cells. Based on these findings, a newly developed ARB, fimasartan, via regulating immune and inflammatory responses might contribute to providing a promising therapeutic strategy for atherosclerotic progression.

## Additional file

Additional file 1: **Table S1.** *In vitro* T cell proliferation assay using CD3^+^ T cells from spleens of ApoE KO mice. **Figure S1.** *In vitro* T cell proliferation assay using CD3+ T cells from spleens of ApoE KO mice. **Figure S2.** Effects of fimasartan on inflammatory cytokines/chemokine expression after CA injury. (DOCX 108 kb)

## Data Availability

All data generated or analysed during this study are included in this published article and its supplementary information files.

## Abbreviations

Ang II: Angiotensin II
ApoE: KO Apolipoprotein E knockout
ARBs: Angiotensin II type 1 receptor blockers
AT1: Angiotensin II type 1
CA: Carotid artery
HDL: High-density lipoprotein
IL: Interleukin
LDL/VLDL: Low-density lipoprotein/Very- Low-density lipoprotein
MMP: Matrix metalloproteinase
SMC: Smooth muscle cell
TGFβ: Transforming growth factor β
Treg: Regulatory T

## References

[CR1] Angeli F (2018). PK/PD evaluation of fimasartan for the treatment of hypertension current evidences and future perspectives. Expert Opin Drug Metab Toxicol.

[CR2] Arishiro K (2007). Angiotensin receptor-1 blocker inhibits atherosclerotic changes and endothelial disruption of the aortic valve in hypercholesterolemic rabbits. J Am Coll Cardiol.

[CR3] Chi YH (2013). Pharmacological characterization of BR-A-657, a highly potent nonpeptide angiotensin II receptor antagonist. Biol Pharm Bull.

[CR4] Choi ET (2005). Matrix metalloproteinase-9 modulation by resident arterial cells is responsible for injury-induced accelerated atherosclerotic plaque development in apolipoprotein E-deficient mice. Arterioscler Thromb Vasc Biol.

[CR5] Dimayuga PC (2011). Enhanced neointima formation following arterial injury in immune deficient Rag-1−/− mice is attenuated by adoptive transfer of CD8 T cells. PLoS One.

[CR6] George J (2012). Regulatory T cells and IL-10 levels are reduced in patients with vulnerable coronary plaques. Atherosclerosis.

[CR7] Hansson GK, Libby P (2006). The immune response in atherosclerosis: a double-edged sword. Nat Rev Immunol.

[CR8] Henderson EL (1999). Death of smooth muscle cells and expression of mediators of apoptosis by T lymphocytes in human abdominal aortic aneurysms. Circulation.

[CR9] Hoch NE (2009). Regulation of T-cell function by endogenously produced angiotensin II. Am J Physiol Regul Integr Comp Physiol.

[CR10] Johnstone MT (2004). Angiotensin receptor blockade with candesartan attenuates atherosclerosis, plaque disruption, and macrophage accumulation within the plaque in a rabbit model. Circulation.

[CR11] Kim S (2015). Fimasartan, a novel angiotensin-receptor blocker, protects against renal inflammation and fibrosis in mice with unilateral ureteral obstruction: the possible role of Nrf2. Int J Med Sci.

[CR12] Kim TW (2012). Synthesis and antihypertensive activity of pyrimidin-4(3H)-one derivatives as losartan analogue for new angiotensin II receptor type 1 (AT1) antagonists. Bioorg Med Chem Lett.

[CR13] Kyaw T (2013). Cytotoxic and proinflammatory CD8+ T lymphocytes promote development of vulnerable atherosclerotic plaques in apoE-deficient mice. Circulation.

[CR14] Lee HW (2011). Effect of age on the pharmacokinetics of fimasartan (BR-A-657). Expert Opin Drug Metab Toxicol.

[CR15] Lee JY (2013). Antiatherosclerotic effects of the novel angiotensin receptor antagonist Fimasartan on plaque progression and stability in a rabbit model: a double-blind placebo-controlled trial. J Cardiovasc Pharmacol.

[CR16] Lee SE (2012). Efficacy and tolerability of fimasartan, a new angiotensin receptor blocker, compared with losartan (50/100 mg): a 12-week, phase III, multicenter, prospective, randomized, double-blind, parallel-group, dose escalation clinical trial with an optional 12-week extension phase in adult Korean patients with mild-to-moderate hypertension. Clin Ther.

[CR17] Liverani E (2014). LPS-induced systemic inflammation is more severe in P2Y12 null mice. J Leukoc Biol.

[CR18] Mallat Z (1999). Protective role of interleukin-10 in atherosclerosis. Circ Res.

[CR19] Mallat Z (2003). Induction of a regulatory T cell type 1 response reduces the development of atherosclerosis in apolipoprotein E-knockout mice. Circulation.

[CR20] Matsumura T (2011). Telmisartan exerts antiatherosclerotic effects by activating peroxisome proliferator-activated receptor-gamma in macrophages. Arterioscler Thromb Vasc Biol.

[CR21] Mehta PK, Griendling KK (2007). Angiotensin II cell signaling: physiological and pathological effects in the cardiovascular system. Am J Phys Cell Physiol.

[CR22] Meir KS, Leitersdorf E (2004). Atherosclerosis in the apolipoprotein-E-deficient mouse: a decade of progress. Arterioscler Thromb Vasc Biol.

[CR23] Meng K (2015). Valsartan attenuates atherosclerosis via upregulating the Th2 immune response in prolonged angiotensin II-treated ApoE(−/−) mice. Mol Med.

[CR24] Meng X (2016). Regulatory T cells in cardiovascular diseases. Nat Rev Cardiol.

[CR25] Newby AC (2005). Dual role of matrix metalloproteinases (matrixins) in intimal thickening and atherosclerotic plaque rupture. Physiol Rev.

[CR26] Pasare C, Medzhitov R (2003). Toll pathway-dependent blockade of CD4+CD25+ T cell-mediated suppression by dendritic cells. Science.

[CR27] Potteaux S, Ait-Oufella H, Mallat Z (2015). Role of splenic monocytes in atherosclerosis. Curr Opin Lipidol.

[CR28] Rezende AB (2011). Splenectomy increases atherosclerotic lesions in apolipoprotein E deficient mice. J Surg Res.

[CR29] Robertson AK (2003). Disruption of TGF-beta signaling in T cells accelerates atherosclerosis. J Clin Invest.

[CR30] Sanchez-Lemus E (2008). Angiotensin II AT1 receptor blockade decreases lipopolysaccharide-induced inflammation in the rat adrenal gland. Endocrinology.

[CR31] Sanchez-Lemus E (2009). Angiotensin II AT1 blockade reduces the lipopolysaccharide-induced innate immune response in rat spleen. Am J Physiol Regul Integr Comp Physiol.

[CR32] Schuett H, Luchtefeld M, Grothusen C, Grote K, Schieffer B (2009). How much is too much? Interleukin-6 and its signalling in atherosclerosis. Thromb Haemost.

[CR33] Spitz C (2016). Regulatory T cells in atherosclerosis: critical immune regulatory function and therapeutic potential. Cell Mol Life Sci.

[CR34] Tao L (2016). IL-35 improves Treg-mediated immune suppression in atherosclerotic mice. Exp Ther Med.

[CR35] Wagsater D, Zhu C, Bjorkegren J, Skogsberg J, Eriksson P (2011). MMP-2 and MMP-9 are prominent matrix metalloproteinases during atherosclerosis development in the Ldlr(−/−)Apob(100/100) mouse. Int J Mol Med.

[CR36] Weber C, Zernecke A, Libby P (2008). The multifaceted contributions of leukocyte subsets to atherosclerosis: lessons from mouse models. Nat Rev Immunol.

[CR37] Yabluchanskiy A, Ma Y, Iyer RP, Hall ME, Lindsey ML (2013). Matrix metalloproteinase-9: many shades of function in cardiovascular disease. Physiology (Bethesda).

[CR38] Zhou HF (2013). CD43-mediated IFN-gamma production by CD8+ T cells promotes abdominal aortic aneurysm in mice. J Immunol.

