# Supplementary material for: Fimasartan reduces neointimal formation and inflammation after carotid arterial injury in apolipoprotein E knockout mice
Source: Mol Med. 2019 Jul 15;25:33. doi: 10.1186/s10020-019-0095-0 (PMC6632006; doi:10.1186/s10020-019-0095-0)
Supplement: Supplementary file 1 — Table S1. In vitro T cell proliferation assay using CD3+ T cells from spleens of ApoE KO mice. Figure S1. In vitro T cell proliferation assay using CD3+ T cells from spleens of ApoE KO mice. Figure S2. Effects of fimasartan on inflammatory cytokines/chemokine expression after CA injury. (DOCX 108 kb) [file 10020_2019_95_MOESM1_ESM.docx]

**Supplementary Information**

**Fimasartan Reduces Neointimal Formation and Inflammation after Carotid Arterial Injury in Apolipoprotein E Knockout Mice**

***Short Title: Effects of Fimasartan after Carotid Arterial Injury***

Jong-Ho Kim^1^, I-Rang Lim^1^, Hyung Joon Joo^1^, Chi-Yeon Park^1^, Seung-Cheol Choi^1^, Han Saem Jeong^1^, Soon Jun Hong^1*^

^1^Department of Cardiology, Cardiovascular Center, Korea University College of Medicine, Seoul 02841, Republic of Korea

Contents:

Supplementary Table (Table S1)

Supplementary Figure and Figure Legend (Figure S1~2)

**Supplementary Table**

|  | **Group I** | **Group II** | **Group III** | **Group IV** | ***p*-value** |
| --- | --- | --- | --- | --- | --- |
| **Total cholesterol**  **(mg/dL)** | **132.64±11.59** | **135.65±19.82** | **147.48±10.72** | **134.63±1025** | **0.089** |
| **LDL/VLDL cholesterol (mg/dL)** | **89.34±17.44** | **65.07±17.51** | **94.80±13.92** | **83.56±10.64** | **0.053** |
| **HDL cholesterol**  **(mg/dL)** | **47.60±14.25** | **51.81±13.07** | **43.95±9.36** | **38.20±9.08** | **0.078** |
| **Triglycerides**  **(μg/dL)** | **84.58±11.12** | **76.63±14.27** | **87.53±15.18** | **83.55±7.17** | **0.410** |

**Table S1. *In vitro* T cell proliferation assay using CD3^+^ T cells from spleens of ApoE KO mice.** (A) Representative pictures of flow cytometry 5 days after treatment without or with fimasartan to CD3^+^ T cells. (B) Quantification by measuring CFDA SE signal. Values are presented as the mean ± SD. **p* < 0.05.

**Supplementary Figure and Figure Legend**

**
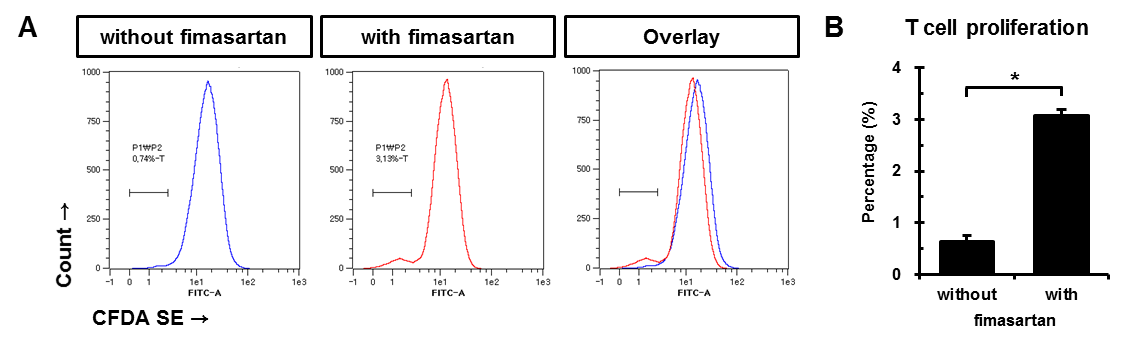
**

**Figure S1.**  ***In vitro* T cell proliferation assay using CD3+ T cells from spleens of ApoE KO mice.** (A) Representative pictures of flow cytometry after 5 days of treatment without or with fimasartan to CD3^+^ T cells. (B) Quantification by measuring CFDA SE signal. Values are presented as the mean ± SD. **p* < 0.05.


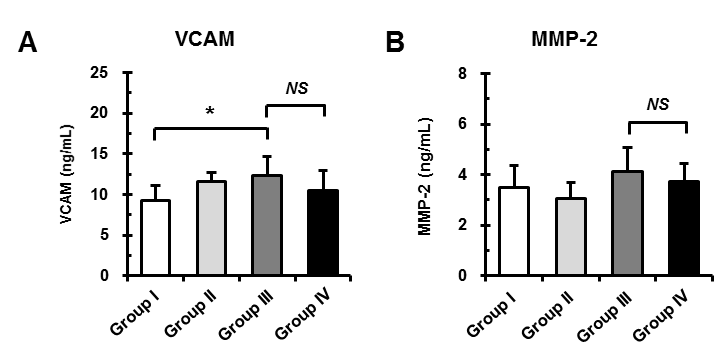


**Figure S2. Effects of fimasartan on inflammatory cytokines/chemokine expression after CA injury.** Plasma levels of VCAM (A) and MMP-2 (B) in peripheral blood. All experimental data are from *n* = 12 of ApoE KO mice in each group. Values are presented as the mean ± SD. **p* < 0.05. *NS*, not significant.
